# A profile of lung cancer in the young population with a highlight on the Indian perspective

**DOI:** 10.3389/fonc.2025.1614463

**Published:** 2025-09-01

**Authors:** Ghazal Tansir, Aparna Sharma, Sachin Khurana, Deepam Pushpam, Prabhat Singh Malik

**Affiliations:** Department of Medical Oncology, All India Institute of Medical Sciences, New Delhi, India

**Keywords:** lung cancer, young onset cancer, personalized medicine, oncofertility, targeted therapy

## Abstract

Non-small cell lung cancer (NSCLC) is a disease primarily of the elderly, with a small proportion of patients in the younger age group. This subgroup of younger patients accounts for 1-10% among the Asian population and 2% in Caucasians. While variable age cut-offs have been taken for studies among these patients, there is sparse knowledge about the unique predisposing factors and etiology of lung cancer arising in them. Prior studies suggest that genetic factors, including Mendelian inheritance patterns and germline mutations, may contribute to early-onset lung cancer. Additionally, shorter durations of tobacco exposure in younger patients raise questions about alternative etiologies. Thus, there is potential for further research into the role of pathogenic germline mutations such as of the BRCA1, BRCA2 and TP53 genes. The higher prevalence of targetable genomic alterations such as EGFR mutations, ALK and ROS1 fusions in the young, and the lower proportion of BRAF, KRAS and MET alterations has therapeutic implications. Therapeutic outcomes among younger patients with lung cancer in localized and metastatic settings in real-world studies have been shown to be better than their older counterparts. It is notable that very young patients (less than 30 years of age) may have worse biology than those a decade older. Clinical trials assessing targeted treatments with tyrosine kinase inhibitors demonstrated equivalent results across age subgroups but representation of younger patients is disproportionate. Survival outcomes with immunotherapy for advanced lung cancer have shown the most improvement in those aged less than 55 years. Hence, treatment outcomes remain a subject of interest within this specific population, along with the issues of fertility, cancer treatment during pregnancy, financial toxicity and psychosocial counseling. There is paucity of literature on young Indian patients with lung cancer despite them presenting a decade earlier than the global population. Further studies are needed focusing on driving mutations, genetic, environmental and demographic factors influencing the presentation and treatment outcomes among Indian patients. This review focuses on the knowledge that exists and that which needs to be generated on these issues on young patients with lung cancer, with a spotlight on the Indian setting.

## 1 Introduction

Lung cancer is the most common cancer globally consisting of predominantly non-small cell lung cancer (NSCLC), diagnosed at a median age of 70 years (1–3). However, a small proportion of patients are now presenting at a younger age, the cut-off of which has been variably described (4–6). Less than 2% of patients in the Caucasian and less than 5% in the Chinese population respectively with NSCLC are aged less than 45 years (4, 5). Other studies have described 1-10% of newly diagnosed patients with NSCLC to be less than 40 years of age (7, 8). While previous studies over the years have explored the risk factors, etiology and epidemiology of NSCLC, young-onset lung cancer (YLC) remains an under-described subset. There is sparse literature on the unique disease biology, targetable genomic alterations, treatment options and effectiveness in young patients with lung cancer. Moreover, there are issues pertaining to oncofertility, lung cancer in pregnancy, psychological aspects and discussions on early death that clinicians should be apprised with. Existing research in other malignancies, such as breast and colon cancer, has been performed to study genetic and familial factors influencing the disease onset (9, 10). Similarly, NSCLC in young patients may also represent a biologically distinct subset of the disease, harboring potentially targetable genomic alterations. However, studies focusing specifically on the frequency and spectrum of these alterations among young patients with NSCLC are limited. Studies on treatment outcomes have also yielded variable results and there is developing evidence of aggressive disease behavior in this population (11). India comprises a unique profile of patients with lung cancer considering the age of onset is a decade lower than the global statistics, per data obtained from our center’s lung cancer clinic (12). Demographic dynamics may play a role in this observation with a greater proportion of the Indian population aged in the bracket of 15–64 years, but a thorough exploration of more complex interactions is warranted (13). Given the emerging landscape of personalized medicine, a comprehensive understanding of the genomic and clinical characteristics in this population is crucial. We hereby present a profile on young patients with NSCLC covering the risk factors, genetic mutations, treatment outcomes and special considerations to encapsulate their distinct biological behavior and their unique therapeutic implications. We also throw light on the existing, albeit sparse information on Indian patients with lung cancer, belonging to the younger subset.

## 2 Extrinsic factors explored for the development of lung cancer in the young

Exposure to tobacco smoke has been a well-established primary risk factor for lung cancer in a majority of cases (14). However, the prevalence of smoking among patients with YLC is lower than older patients and for a shorter duration of time. Among Asians, only 30% of patients with YLC are former or current smokers, and duration of exposure to tobacco smoke is also lower at 12 years compared to 49 years among Europeans (15, 16). A recently published Indian study by Malik et al. from two tertiary care centers among 133 YLC patients (aged less than 40 years) found a prevalence of smoking or tobacco use in approximately 21% patients only (17). Therefore, multiple other factors could also be contributory to the development of cancer in these young smokers, namely second-hand smoking and environmental radon exposure (18, 19). Second-hand smoke exposure remains a formidable concern in the Indian setting, exhibiting a high prevalence in home and workplace settings despite the fact that smoking is banned in public areas since 2003 (20). Domestic exposure to second-hand smoke also may contribute to the rising incidence of lung cancer among Indian women, as described by Noronha et al. (21).

Outdoor air pollution exposure is also associated with an elevated risk of lung cancer and 14% of deaths related to the disease (22, 23). Pre-clinical data links the development of drivers such as EGFR and KRAS mutations and cancer-promoting behavior due to exposure to fine particles ≤2.5 μm (24). The relative risk of air pollution-related lung cancer among patients aged less than 50 years is higher (1.63) as compared to those aged 50-64 (1.11) and greater than 65 (1.15) years respectively. Therefore, outdoor air pollution has been classified as a group I carcinogen for development of lung cancer (25). Meanwhile, indoor air pollution exposure to cooking fuel remains a risk factor among Indian women, though its use is slowly reducing in current times (26, 27). A district-level study conducted among the rural region of Nagpur in India between 2016–18 found the use of biomass fuel to be prevalent in 88.7% of households, owing to factors such as low household incomes, lack of separate kitchen areas as well as a continued behavioral preference (27). Exposure to non-tobacco factors such as air pollution or poor environmental air quality was also considered as an attributable risk factor by Iyer et al, who found a lower prevalence of smoking in Indian female patients with lung cancer as compared to their male counterparts (28).

## 3 Genetic and familial factors explored for the susceptibility to lung cancer in the young

While extrinsic factors such as the above are implicated in YLC, genetics and familial causes are also emerging risk factors. Studies have examined the possibility of a genetic component to the disease especially when the onset occurs at an early age. Genome-wide association studies (GWAS) initially attempted to identify genetic factors related to development of YLC. Multiple cancer susceptibility genes have been found to correlate according to subgroups of lung cancer such as by ancestry, exposure to environmental carcinogens, and those associated with multiple cancer sub-sites (29). For instance, an association with smoking has been found on interacting loci at chromosomes 14q22.1 and 15q22.32 (30). Similarly, loci for asbestos-related lung cancer have been found at chromosomes 2q34, 7q32.1, 11q13 and 22q13.31 (31, 32). 5p15.33 is a multi-cancer locus which has been associated with telomere biology, having the most robust signal at rs2736100 (33). Pathways related to the major histocompatibility complex region have been found independently associated with loci at chromosomes 6p21.33 and 6p21.32 for squamous cell and adenocarcinomas respectively (34, 35).

Familial aggregation studies have found a higher risk of lung cancer onset at earlier than 55 years of age among those with any lung cancer-affected relative(s) (36). Among patients with lung adenocarcinoma included in The Cancer Genome Atlas (TCGA), 2.5-4.5% patients possessed germline variants that could be linked to a risk of cancer as part of Mendelian syndromes (37). However, the mean age of this dataset was 65.3 years (range 33-88) and patients aged less than 40 years comprised only 7% of the total patients included. A meta-analysis conducted by the Internal Lung Cancer Consortium consisting of 24000 cases and 23000 controls has provided important findings in the form of a 1.5-fold increase in risk of lung cancer related to a significant family history (38). The genetic component could be associated with certain pathogenic germline variants (PGVs) especially those involved in DNA repair pathways (39). Wei et al. demonstrated the high prevalence of pathogenic/likely pathogenic (P/LP) variants among patients with lung cancer especially in YLC (40). The odds ratio of 4.1 with BRCA1 and 29.2 with TP53 showed that the P/LP variants of these genes are associated with risk of lung cancer. Exome sequenced samples obtained from female never-smokers younger than the age of 45 years found 8 potentially heterozygous mutations including BRCA1 variant p.Cys47Arg, BRCA2 variant p.Arg2784Trp and COL6A1 (41). There is also the suggestion from the LIFESCREEN randomized controlled trial that lung cancer could be a part of the Li Fraumeni Syndrome cancer spectrum, though it is not classically included in the same (42). The age of onset of lung cancer and concept of accelerated onset of disease in patients with germline pathological variants (PVs) was also explored by the analysis of a retrospective cohort (43). Building an accelerated failure time model, the authors found that presence of PVs advanced the onset of lung adenocarcinoma. BRCA2, TP53 and other Fanconi Anemia genes advanced the age of lung cancer onset by 12.2 (95% confidence interval [CI], 2.5-20.6), 9.0 (95% CI, 0.5-16.5) and 6.1 (-1-12.6) years.

Malik et al. observed that among YLC Indian patients, 9.5% patients had a family history of cancer (defined by them as any cancer present in 1 first-degree or 2 second-degree relatives) (17). Another Indian study conducted at our center screened 570 Indian patients with NSCLC and found 78 patients who had at least 1 affected first-degree or 2 affected second-degree relatives with any malignancy. Among this cohort of 78 patients (median age 60 years), 13 (17%) had P/LP germline variants consisting of BRCA1 (n=1), BRCA2 (n=2), CHEK2 (n=1), ATM (n=2), BAP1 (n=1), FANCA (n=1), FANCI (n=1), FANCM (n=1), LZTR1 (n=2) and XRCC3 (n=1) (44). These findings suggest potential heritable mechanisms that may predispose individuals to early lung cancer, especially in low-exposure populations. There is a scope for large familial segregation studies and germline mutation analysis, that could identify susceptible genes and effective screening strategies. **Table 1** describes the various studies (45–58) that have explored familial aggregation and germline mutations as potential factors in patients with YLC.

**Table 1:** Summary of studies describing familial aggregation and genetic susceptibility among young patients with lung cancer.

S. no.	Study (by year of publication)	Study population (Race/ethnicity composition)	Age cut-off for definition of young patient	Number of patients included	Results
Familial aggregation studies					
1.	Schwartz et al (45) (1996)	Case-control study among non-smokers aged 40–89 years (Caucasian)	59 years	257 cases 277 controls	7.2-fold increase in risk of cancer with first-degree relative having lung cancer in age group 40–59 years
2.	Kreuzer et al (46)(1998)	Case-control study (European)	45 years for defining young patients 55–69 years for defining old patients	Young group: 251 cases, 280 controls Older group: 2009 cases 2039 controls	2.6-fold (95% CI: 1.1-6.0) increase in risk of lung cancer in young age group with history of lung cancer in first-degree relative No such increase in older age group
3.	Schwartz et al (47) (1999)	Case-control study included young patients with lung cancer and their first-degree female relatives (Caucasian)	40 years	118 cases 161 controls	History of early-onset lung cancer elevated breast cancer risk in first-degree relative by 5.1-fold (95% CI: 1.7-15.1)
4.	Hemminki et al (48) (2005)	Population included in Swedish Cancer Registry Database including patients with lung cancer with family history of lung cancer (European)	50 years	5290	1.7% lung cancers up to the age of 68 possible heritable, probably due to a high-penetrant recessive gene(s) that predispose to tobacco carcinogens
5.	Coté ML et al (49) (2005)	Case-control study on patients with lung cancer and their families (Caucasian)	50 years	692 cases 773 controls	Relatives of black cases had significantly higher risk of lung cancer compared to white cases (OR 2.07, 95% CI: 1.29-3.32)
6.	Cassidy et al (50)(2008)	Case-control study on patients with lung cancer and non-cancer population controls (British)	60 years	579 cases 1151 controls	Significantly elevated risk of lung cancer with affected relative <60 years (OR 2.08 95%CI 1.2-3.59); higher risk when incident case was < 60 years (OR 4.89 95%CI 1.47-16.25)
S.No.	Study (by year of publication)	Study population (Race/ethnicity composition)	Age cut-off for definition of young patient	Number of patients included	Results
Genetic susceptibility studies					
1.	Taioli et al. (51) (2003)	Subgroup analysis from the GSEC database	45 years	261 cases 1452 controls	Significant association between lung cancer and GSTT1 null genotype (OR = 1.2; 95% CI: 1.0–1.6), and homozygous CYP1A1 Msp1variant allele (CYP1A1*2A and *2B) genotype (OR = 4.7 95% CI: 1.2–19.0).
2.	Coté ML et al. (52)(2005)	Case control study from SEER database (Caucasian – 65.4%, Black – 34.6%)	50 years	350 cases 410 controls	African-Americans carrying at least one G allele at the GSTP1 locus were 2.9-fold more likely to have lung cancer (95% CI 1.29-6.20).
3.	Landi et al. (53) (2006)	Case control study with lung cancer cases and matched controls (European)	50 years	299 cases 317 controls	Significant associations with lung cancer and polymorphisms in genes involved in DNA damage sensing (ATM) and, in four genes encoding proteins involved in mismatch repair (LIG1, LIG3,MLH1, and MSH6) found.
4.	Gemignani F et al. (54) (2007)	Case control study from Europe (European)	50 years	299 cases 317 controls	Heterozygote carriers of SNPs in CYP1A2 1545T.C, 164C.A and 740T.G; CYP2A6 47A.C; MDR1 3435T.C; NAT1 1088T.A and 1095A.C;GSTA2 S112T; GSTM3 V224I and MTHFR A222V had altered risk of developing lung cancer. NAT1 fast + NAT2 fast acetylator phenotypes were at lower risk
5.	Rosenberger et al. (55) (2008)	Case control study from Germany (European)	50 years	246 cases 233 controls	Carriers of Leu-allele of GPX1(Pro200Leu) showed significant risk reduction of OR = 0.6 (95% CI: 0.4–0.8, p = 0.002) in general and of OR = 0.3 (95% CI:0.1–0.8, p= 0.012) in heavy smokers. Risk decreasing genetic effect for His-carriers of EPHX1(His113Tyr) for moderate smokers (OR = 0.2, 95% CI:0.1–0.7, p = 0.012).
6.	An et al. (56) (2008)	Hospital-based Chinese study with lung cancer cases and non-cancer controls (Asian)	59 years	500 cases 517 controls	Overall population: rs1799977 I219V polymorphism marginally associated with risk of lung cancer (P = 0.055). Stronger association in younger patients (OR 5.28; 95% CI 1.45—19.21).
7.	Sauter et al. (57) (2008)	Case control study with lung cancer cases and matched controls (European)	51 years	635 cases 1300 controls	Several SNPs significantly associated with early-onset lung cancer. Most significant effect for rs1938901 (p= 0.0089), rs193008 (p= 0.0108), and rs996999 (p= 0.0459).
8.	Graziano et al. (58) (2009)	Case control study with lung cancer cases aged less than 45 years and controls aged more than 60 years (European)	45 years	24 cases 71 controls	Significant association between early-onset lung cancer and the EPHX1 exon 4 variant (OR = 3.33, 95% CI = 1.50–7.41).

CI, Confidence interval; OR, Odds ratio; GSEC, Genetic Susceptibility to Environmental Carcinogens; SEER, Surveillance; epidemiology and end results; SNP, Single-nucleotide polymorphisms.

## 4 Clinico-epidemiologic features unique to young patients with lung cancer

### 4.1 Gender distribution

Various studies have demonstrated the higher proportion of female patients among the YLC subset. Thomas et al. described patients with lung cancer younger than 40 years using the surveillance, epidemiology and end results (SEER) with a numerically though statistically non-significant predominance of the female population (51% versus 49%) (59). These findings contrasted with the gender distribution among patients aged more than 40 years, in whom the representation of males was significantly higher. Similar results were found by Suidan et al, who reported a statistically non-significant female predominance in the cohort of patients aged up to 50 years versus those above 60 years (60). SEER registry data in the 2010 study by Subramanian et al. demonstrated a significant difference in the proportion of females in the YLC population (48.7% versus 41.9) (61). These results have been mirrored by findings from Indian studies also, with Malik et al. reporting the percentage distribution of female patients in the YLC cohort to be 41% (17). A significant difference between the proportion of female patients in the cohort of patients aged less than 40 versus more than 60 years of age was reported by Vashistha et al. (62). This comparison conducted between 154 YLC patients and 1058 older patients found 26% versus 14.5% patients in the respective groups with a statistically significant difference between the two. Though the proportion of females found in their YLC cohort is lesser than that reported in other Indian and Western studies, the difference with the older subgroup throws important lights on the gender distribution. The smoking habits have exhibited interesting trends with Sekine et al. finding that male patients had a lower proportion of young current or former smokers (84%) vis-a-vis the older subgroup (95%). In contrast, female patients had equal (39%) prevalence of smoking, though the older subgroup had a higher composition of heavy (more than 40 cigarettes per day) (63).

### 4.2 Clinical presentation and radiological findings

The most common symptoms include cough (30%), chest pain (8%), hemoptysis (7%), metastatic symptoms such as bone pains or neurological complaints (6% each). Notably, a few cases (10%) may well be discovered incidentally (60). A majority among the patients of YLC present at advanced stages upon diagnosis. The range of patients who had stage IV disease at presentation varies from approximately 50%-70% as described in various studies (64, 65). While the percentage distribution may vary in terms of statistical significance, there are studies which exhibit a significantly high prevalence of stage IV disease among the YLC cohort. It has also been shown that the patients harboring stage I disease were significantly higher in older subsets as compared to those in the YLC group (20.9% versus 11.7%) (60). The metastatic pattern of disease spread has been shown to have significant distinctions such as in brain (18.5% versus 9.5%), liver (7.1% versus 4.6%) and bone (16.2% versus 11.7%) between the YLC and older cohort respectively (66). The YLC group harbors a significantly higher proportion of upfront brain metastasis during their entire disease course (39% versus 25%) and among them, greater than 2 brain metastases have been found in almost one-fifth patients (60). A study conducted at an Indian tertiary care center described the symptomatology of lung cancer to comprise cough (81%), loss of appetite (66%), dyspnea (54%) and fatigue (60%) among others (67). Though this study did not recruit only YLC patients, it compared smokers versus non-smokers in their cohort. The non-smokers consisted of 64% patients aged less than 60 years of age and had a greater proportion of presentation at advanced stage (78.7%) as compared to the smoker cohort (59.7%).

The Indian setting poses diagnostic challenges and delays in patients with lung cancer due to the high endemicity of pulmonary tuberculosis. The overlapping clinical and/or radiological features may lead to misdiagnosis in greater than 40% patients, causing stage progression and worse survival outcomes due to diagnostic delays (68). The overlapping clinical and/or radiological features may lead to misdiagnosis in greater than 40% patients, causing stage progression and worse survival outcomes due to diagnostic delays (69, 70). The study by Malik et al. in contrast, consisted of YLC patients exclusively and found a much higher proportion of patients with stage IV disease at 91.7% including 64% with multi-site metastases (17). The prevalence of brain metastases at 40% in this study also corroborated with findings of Suidan et al. (60). Further description of studies with details on clinico-pathological findings in patients with YLC are presented in **Table 2**.

**Table 2:** Summary of studies presenting clinico-pathological details of young patients with lung cancer.

S. no.	Study (by year of publication)	Age cut-off for definition of young patient	Number of patients (Race/ethnicity composition of study population)	Clinico-pathological differences	Survival outcome differences
1.	De Caro et al. (71) (1982)	40 years	35 (Caucasian)	48.6% adenocarcinoma and 28.6% small cell undifferentiated cancers in young patients	Young patients not operated had shorter OS compared to older patients (p<0.0001); no difference in those who underwent surgery
2.	Nugent et al. (72) (1997)	45 years	55 young patients 108 elderly (greater than 80 years of age) patients	6% young patients had stage I/II disease; 32% operated 33% elderly had stage I/II disease; 6% operated	Longer OS for young patients compared to elderly (Stage I-IIIA) (p<0.05); no difference in stage IIIB and beyond
3.	Sekine et al. (63)(1999)	40 years	91 (Asian)	Male patients: 84% smokers in young, 95% in old; 71% adenocarcinoma in young; more advanced disease (75%) in young Female patients: 39% smokers in both young and old groups; 92% adenocarcinoma in young	Equivalent OS between young and old age groups in both males and females
4.	Etzel et al. (73) (2006)	50 years	230 young patients 426 elderly (aged more than 70 years) patients (Caucasian)	Higher proportion of never-smokers (23.9%) and former smokers (59.2%) in young patients compared to 17.6% and 17.8% respectively in older group; Adenocarcinoma predominated in young patients (55.2%) 83.4% young patients presented with stage III/IV disease compared to 58.6% older patients	Median OS 16.7 months in young versus 19.2 months in older group Female young patients had longer OS (20.7 months) versus males (13 months), p=0.004
5.	Subramanian et al. (61) (2010)	40 years	2775 (Caucasian – 82.7%, African-American – 10.9%, Asian – 5.9%)	Significantly more young African-American patients (19.2% versus 10.9%), Asians (10.3% versus 5.9%), women (48.7% versus 41.9%), Stage IV presentation (57.4% versus 43%), adenocarcinomas (57.5% versus 45.2%) compared to older	Better 5-year OS in younger patients versus older across stages
6.	Zhang et al. (8) (2010)	45 years	652 (Asian)	Higher adenocarcinomas in young male patients compared to older group (63.7% versus 43.1%)	Median OS of young patients was significantly shorter than middle-aged patients (45–60 years) but similar to older group (more than 60 years of age)
7.	Hsu et al. (74) (2012)	45 years	144 (Asian)	51.4% female patients Adenocarcinomas predominate in young males (77.1%) and females (87.8%)	Young males had shorter OS (HR 1.70, 95% CI 1.08-2.68) compared to females
8.	Thomas et al. (59) (2015)	40 years	2786 (Caucasian – 69.5%, Black – 15%, American-Indian – 0.65%, Asian – 14.3%, Other – 0.36%)	51% female patients 68% presented with advanced disease	Young patients had better median all-cause and lung cancer-specific survival compared to older patients for localised (not reached versus 46 months), regional (28 months versus 17 months), metastatic (9 months versus 5 months) diseases p<0.001
9.	Rich et al (6) (2015)	40 years	651 (British)	48% adenocarcinomas Similar distribution of stage of disease (71% with Stage IIIB and beyond) Young group was more likely to receive surgery and chemotherapy	Young patients had better OS and post-operative survival compared to older patients
10.	Arnold et al. (75)(2016)	46 years	5657 (Caucasian – 87%, Hispanic – 4%, Others – 9%)	Female predominant, non-white ethnicities, advanced disease at presentation among younger patients	Absolute differences in OS among young versus older patients was 25%, 9%, 2% for stages I, II, III/IV respectively
11.	Hsu et al. (76) (2016)	45 years	1074 (Asian)	48.4% females in younger group versus 38.2% in older (p<0.001); 47.3% never-smokers in younger group versus 43.8% in older (p=0.015); 70.4% adenocarcinomas in younger group versus 58.1% in older (p<0.001)	Outcomes not reported
12.	Malik et al. (17) (2025)	40 years	133 (Indian)	40.6% females 85.7% adenocarcinoma 91.7% had stage IV disease	Median OS = 26 months (95% CI: 15.56-32.43); Subset aged less than 30 years: Median OS = 15.67 months (95% CI: 5.86-30.03)

CI, Confidence interval; HR, Hazard ratio; OS, Overall survival.

## 5 Histology and molecular landscape of patients with young lung cancer

The most common histological subtype among more than 75% patients with YLC is adenocarcinoma, a proportion significantly higher than the older subset (65, 66). **Table 3** provides details of studies that describe the histopathological subtyping for patients with YLC.

**Table 3:** Summary of studies with description of genomic alterations including therapeutic targets among young patients with lung cancer.

S. no.	Study (by year of publication)	Age cut-off for definition of young patient	Number of patients (Race/ethnicity composition of study population)	Methodology	Results
1.	Sacher et al. (11) (2016)	50 years	340 (Caucasian-90%, Asian-4%, Black-4%, Hispanic-1%)	Sequencing on FFPE samples	Younger age associated with EGFR (p=0.02) and ALK (p<0.01) alterations Younger age associated with targetable GA (p<0.001) Targetable GA improved survival; young and old patients had similar outcomes in absence of targetable GA
2.	Vavalà et al. (77) (2017)	45 years	26 young patients 21 old patients 21 healthy young controls (European)	Targeted NGS of young versus old patients on archived FFPE tissue	Higher proportion of TP53 and EGFR (mostly exon 19 deletion) in young patients; TP53 hotspots: R248, R273, G245, R282 Higher KRAS variations in old
3.	Tanaka et al. (78) (2017)	40 years	81 (Asian)	Sequencing on FFPE samples	ALK translocation (41%) EGFR mutations (30%) KRAS mutations (2%) Young patients having adenocarcinoma without GA associated with worse overall survival compared to old patients without GA (8.9 versus 16.4 months, p<0.001)
4.	Donner et al. (41) (2018)	45 years	21 female patients (European)	WES of germline DNA using archival normal tissue material	Potentially pathogenic variants in 8 Cancer Gene Census germline genes: BRCA1, BRCA2, ERCC4, EXT1, HNF1 A, PTCH1, SMARCB1 and TP53. Variants in TP53, BRCA1, and BRCA2 likely led to early onset of disease (in 14%).
5.	Luo et al. (79) (2018)	45 years	36 (Asian)	WGS of tumor tissue and normal lung tissue	Germline mutations in BPIFB1 (rs6141383, p.V284M), CHD4 (rs74790047, p.D140E), PARP1 (rs3219145, p.K940R), NUDT1 (rs4866, p.V83M), RAD52(rs4987207, p.S346*), and MFI2 (rs17129219, p.A559T) significantly enriched in the young never-smoker patients compared with the in-house noncancer database (p < 0.05)
6.	Hou et al. (15) (2018)	45 years	87 young and 90 older patients (Asian)	Targeted NGS of surgically resected tissue in both groups	Higher frequency of ALK and HER2 alterations. Higher frequency of concurrent EGFR/TP53
7.	Giordano et al. (80) (2018)	50 years	44 (European)	Micro RNA profiling from resected specimen using FFPE samples	7 miRNAs (miR-25-3p, miR-29c-3p, miR-33a-5p, miR-144-3p, miR-153-3p, miR-342-5p and miR-485-3p) higher in younger patients (*P*<0.05). miR-144-3p had an opposite influence on overall survival: upregulation was associated with a worse prognosis in young patients (*P*=0.01) and with a better outcome in the older group (*P*=0.03).
8.	Yang et al. (81) (2019)	36 years	20 young and 24 older (aged more than 52 years) patients (Asian)	WES on FFPE samples	Mutations in FRG1 (p=0.027) and KMT2C (p=0.046) higher in younger patients. Pathogenic germline mutations observed in TP53, TGFBR2, MLH3 and ELAC2 in younger patients. Similar somatic mutations in young and old patients
9.	Chen et al. (82) (2019)	40 years	181 young in a total cohort of 1000 (Asian)	Targeted NGS of 1000 patients	Higher targetable GA in younger patients (p<0.001) Higher prevalence of rare GA in younger patients such as HER2, ROS1, MET (p<0.05) Frequencies of EGFR mutation, ALK rearrangement and KRAS mutations were 34.3%, 37.6%, 6.1% respectively Similar prognosis for young and old patients with GA (p>0.05)
10.	Yang et al. (83) (2019)	40 years	54 (Asian)	Comprehensive genomic profiling	Higher GA in younger (68.5%) versus older (54.8%), p=0.05 Higher fusion genes in younger (22.2% versus 4.1%, p<0.001) Equivalent gene mutations (46.3% versus 45.3%, p=0.92) Similar overall survival (50.2 versus 51.4 months, p=0.112)
11.	Hou et al. (84) (2020)	45 years	177 (Asian)	cBioPortal and NGS analysis	Higher targetable GA in younger patients (p<0.001) Higher EGFR (p=0.005), ALK alteration, ROS1 fusion (p=0.035), RET rearrangement (p<0.001) KRAS (p<0.001) and MET (p=0.057) more in older patients
12.	Malik et al. (17) (2025)	40 years	133 (Indian)	TruSight Oncology 500 panel on FFPE samples	EGFR mutations (35.51%) ALK rearrangement (65.7%) ROS1 rearrangement (7.25%) KRAS mutation (3.7%) TP53 co-mutation (1.5%) ERC1-RET fusion (0.75%)

NGS, Next-generation sequencing; FFPE, Formalin-fixed paraffin-embedded; WES, Whole exome sequencing; GA, Genomic alterations; WGS, Whole genome sequencing; RNA, Ribonucleic Acid; miRNA, Micro Ribonucleic Acid.

The presence of oncogenic driver alterations has been found to be significantly greater in the YLC population as compared to the older adults, accounting for 57-70% cases in the former (11, 85, 86). Sacher et al. demonstrated a statistically significant difference in the prevalence of oncogenic genomic alterations among the YLC subset (68%) compared to 52% in the population older than 40 years. It was also noted that smoking status, female gender and Asian race were associated with the occurrence of a targetable genomic alteration in patients with YLC (11). This finding underscores the importance of comprehensive tumor profiling to enable detection of the actionable alterations for utilization of personalized medicine in this subset. The most frequent alterations in YLC include EGFR mutations, ALK and ROS1 fusions (11, 60) While the rarity of RET and NTRK 1/2/3 fusions has led to relatively scarce data, some alterations such as KRAS and MET mutations are linked to more advanced ages and smoking history (13, 86). **Table 3** compiles the landscape of molecular alterations described in various studies covering the YLC cohort. A brief description of these genomic alterations is further described below:

### 5.1 EGFR mutations

Various studies have yielded different results regarding the prevalence of EGFR mutations in the YLC versus older population subset. Hsu et al. found a significant prevalence of EGFR alterations in patients with YLC compared to older counterparts aged greater than 45 years (60.6% versus 52.5%) (76). Similar outcomes were observed in other studies among Asian patients including that by He et al. wherein YLC patients aged less than 40 years had statistically higher proportion of EGFR mutations in contrast to the older subgroup (87). Multiple analyses also showed a numerically, albeit statistically insignificant higher EGFR alterations in patients with YLC. Moreover, there is a notable difference in the type of EGFR mutations observed in the YLC subset, with the EGFR Deletion 19 being more prevalent as compared to L858R in the older groups (88). In contrast, EGFR Exon 20 insertion is more prevalent in the older group (8% versus 1%) and reaching statistical significance (15).

### 5.2 Gene fusions

It has been demonstrated by Yang et al. that gene fusions are more prevalent in young (23.3%) as compared to the older population (5.9%) with ALK rearrangements being most prevalent (83). Multiple published studies reveal the statistically significant frequency difference between YLC and older population, with ALK rearrangements exceeding in the former (68). EML4 is the most common partner gene for these patients in more than 80% cases as exhibited by Tian et al. (89). ROS1 has also been variably shown to have greater prevalence in the young with Wu et al. finding 6% ROS1 mutations in the YLC population (90). Regarding RET alterations, the data is variable with few studies noting a prevalence ranging between 2.8% to 9.5% (84, 91). The analysis from cBioPortal for Cancer Genomics database showed a significantly higher prevalence of RET rearrangements in the YLC group (9.5%) versus those greater than 45 years (1%) (84).

### 5.3 KRAS and BRAF mutations

These mutations have been reported at higher frequencies in the older populations, as described in the multivariate analysis by Sacher et al. (11). There is a lower incidence of the KRAS mutation in earlier decades as low as 9% in less than 40 years and 30% in the range of 60–69 years.

Among other alterations, HER2 alterations may be more prevalent among patients with YLC, as indicated by two studies that found significant differences compared to older patients. Hou et al. reported a 13.8% HER2 amplification rate in the patients with YLC versus 4.4% in older patients (15). However, there is also coexistent data which has found no age-related differences in HER2 status (60). Research on MET alterations remains limited and inconclusive. One study divided 2025 Chinese patients with lung adenocarcinoma into young (up to 50 years of age), intermediate (51–69 years of age) and aged (greater than 70 years of age) and reported a significant difference in the prevalence of MET alterations. Among these, MET amplification was present in 0.24%, 1.18% and 1.78% while MET Exon 14 skipping mutation was found in 0.72%, 1.1%, 3.25% respectively in the three subgroups (92). Additionally, clinical trials on tepotinib and capmatinib suggested that patients with MET-altered NSCLC were older than those with other genotypes receiving targeted therapies, affecting individuals up to 74 years old (93, 94).

### 5.4 A brief insight of genomic alterations into the Indian population

Malik et al. in their analysis of the Indian YLC cohort found the prevalence of EGFR mutations comparable at 35% to other Asian studies (17, 78). The types of EGFR mutations noted in this population consisted of deletion 19 (52.6%), L858R (31.5%), Exon20insertion (10.5%) and compound mutation (5.2%). At 34.2%, ALK rearrangement was found higher in prevalence compared to data obtained from the cBioportal database at 9.5% among those aged less than 45 years (17, 84). A higher prevalence of driver mutations was also described by Sharma et al. in a study comprising of Indian patients with lung cancer at 80.6%, predominantly TP53 (37%), EGFR (34.1%), KRAS (13.3%) and ALK (8.8%). Upon age-wise analysis of this cohort, ALK (24.4%) and ROS1 (7.5%) fusions were more prevalent in patients aged less than 40 years while KRAS mutations (13.6%) were significantly higher in those aged greater than 40 years (95). The KRAS mutation prevalence among YLC patients at 3.7% described by Malik et al. was also comparable to this data and consisted of G12C (40% of KRAS mutated patients) and G12D (60% of KRAS mutated patients) respectively (17).

## 6 Treatment implications and survival outcomes of patients with YLC

Treatment of the younger population for lung cancer produces variable survival outcomes and responses. Thomas et al. described a marked difference in outcomes of localized disease than for metastatic disease between the age groups. The median overall survival (OS) among patients with YLC with localized disease was not reached while it was 46 months in the older population, exhibiting statistical significance (59). A significant difference was also observed among those with metastatic disease in the same study, with 5-year lung cancer specific survival being 9.7% in the YLC as opposed to 4.2% in the older subset respectively (59). Among other recent studies, Suidan et al. found that 69% of both older and younger patient groups underwent molecular profiling with the latter having more treatment changes based upon the results (60). The YLC group showcased differences in management of both localized and metastatic disease in terms of more lung resections, receipt of targeted therapies and lesser rates of dose reductions. The median survival was numerically higher in the younger patients both overall and with driver mutations, though statistical significance was not demonstrated. The younger cohort also exhibited a better survival (24.5 versus 18 months) compared to the older group with brain metastases. In the first-line setting in Indian YLC patients consisting of 92% with metastatic disease, chemotherapy (60.68%) and TKIs (28.2%) were the common treatment agents utilized, producing a median OS of 26 months (95% confidence interval [CI]: 15.56-30.03) as also reported in Western literature (60). A subset of very young patients (aged less than 30 years) had significantly poorer median OS of 15.67 months (95% CI: 5.86-30.03) compared to the older decade of 31–40 years (26 months, 95% CI: 15.9-40.3) indicating an especially aggressive biology (17).

It is important to administer full-dose therapies and use multimodal strategies in YLC patients because they have a lower burden of comorbidities and are described to have an aggressive profile (11). The better tolerance of multimodal strategies could contribute to the significantly better outcomes in loco-regional disease among younger patients. Despite the greater prevalence of genomic alterations in YLC patients, prospective trials focusing on tyrosine kinase inhibitors (TKIs) targeting EGFR and ALK alterations showed consistent outcomes across age subgroups (96–98). Subgroup analyses of data from the first-line immunotherapy trials in advanced lung cancer show comparable survival results in patients divided per the cut-off of 65 years age (99, 100). Conversely, in a large cohort of 53719 patients, with the increased use of immunotherapy post-2011 the 2-year survival probability improved maximum in patients aged less than 55 years (37.7% to 50.3%) (101). These findings support the hypothesis that younger patients may tolerate and respond better to aggressive, multimodal treatment approaches.

## 7 Special considerations on treating lung cancer in young patients

Treating patients with YLC entails certain difficult decisions and conversations, which can be built upon by having a structured multidisciplinary set-up. Addressal of psychosocial concerns, fear about the disease outcomes and a long-term functional life should be performed while the diagnosis is informed to the patients.

### 7.1 Treatment-related toxicity

Most drug-related side effects are more common in the elderly population such as immune-related adverse events (irAEs), TKI-related interstitial lung disease and fluid retention (102). In patients with YLC, fatigue and endocrine irAEs are more frequent (103, 104). Financial toxicity, and a negative impact on physical, emotional and functional well-being QoL scores have been observed, highlighting economic and mental health challenges in these young patients (105).

### 7.2 Fertility preservation

The discussion of fertility preservation and treatment-related infertility should be discussed by clinicians with the patients with YLC. Patients interested in or even uncertain regarding fertility preservation should receive a multidisciplinary consult with fertility specialists and counselors (106). For those planned for receiving TKIs in the curative setting, there needs to be counseling regarding potential effect on gonadal function and the need for a potentially longer washout period before conception in female patients (107). The metastatic setting may also have discussions about pregnancy when the disease has been in prolonged control. Thus, thorough counseling about life expectancy and risk of recurrence should be informed by the treating clinicians.

### 7.3 Management during pregnancy

Treatment of pregnant patients in the YLC group poses challenges in forms of limited diagnostic procedures, drug safety and fetal toxicity. For diagnosis and staging purposes, ultrasound-guided biopsies and non-contrast magnetic resonance imaging are used instead of computed tomography or positron emission tomography (108). The treatment needs to be tailored according to the phase of pregnancy as well as the patient’s general condition. Surgery can be safely performed across all trimesters of pregnancy, though it should be deferred to the second trimester when the risk of miscarriages is lowest (109). However, chemotherapy is contraindicated in the first trimester (110). Platins (preferably carboplatin) in combination with taxanes or vinca alkaloids can be administered from the second trimester onward, but antimetabolites such as gemcitabine and pemetrexed continue to be teratogenic (110, 111). Though a significant number of lung cancer during pregnancy are associated with targetable genomic alterations such as ALK rearrangements (112), animal studies report post-implantation embryo loss, reduced fetal weight and congenital anomalies with multiple TKIs, hence their use remains contraindicated during pregnancy (113). Similarly, immunotherapy is also avoided during the pregnancy duration because PD-L1 has a role to play in feto-maternal tolerance mechanisms.

### 7.4 Challenges in access to healthcare setups and appropriate treatment in Indian patients with YLC

In the absence of a formal referral system, 45% patients with lung cancer in India end up seeking care of general medicine specialists before they reach the oncology specialists (69). This is significant because among the general physicians, only 27% refer the patients to higher centers for further evaluation. A robust referral system directing patients to specialty setups for timely care and attention is imperative for better management of this rare disease. While data on the referral and healthcare setting access of YLC Indian patients is not available at present, these findings in the overall population with lung cancer highlight the pressing logistic needs of the current Indian healthcare system. Malik et al. also highlight the question of availability of targeted therapies in the Indian YLC patients, as evidenced by the use of osimertinib in only 1 patient (1.5%) among the 63 of those who received TKIs (17). Overall, 20% of the patients in this study with targetable genomic alterations could not receive TKI especially among those with ALK rearrangements as compared to EGFR alterations. While low-cost gefitinib is available in India, ALK TKIs remain higher priced and not covered by public healthcare schemes. For a young population subset containing a high percentage of targetable driver mutations, measures for better access to targeted therapies are urgently warranted.

## 8 Discussion and future directions

This review highlights that YLC represents a biologically and clinically distinct subgroup of NSCLC. Younger patients are more likely to be non-smokers, present with advanced disease, and harbor targetable genomic alterations such as EGFR mutations and ALK/ROS1 fusions. Despite better tolerance to aggressive therapies, this population faces psychosocial, financial, and reproductive challenges that require multidisciplinary support. The literature that covers this unique population of YLC is sparse, but the existing studies we have highlighted in our review display potential areas of dedicated research. There is a need to perform focused studies on comprehensive molecular characteristics, familial aggregation studies and germline mutation testing. While studies have explored the possible familial and genetic linkages pointing to Mendelian inheritance patterns in lung cancer (35, 37), the data remains scarce. Patients with YLC are currently grossly under-represented in large-scale genomics data on lung cancer included in the TCGA and need more attention in future analyses. With better knowledge generated on potential germline factors, strategies on screening and personalized treatment could be developed for patients with YLC.

Studies have shown improved survival outcomes among younger patients especially with the use of targeted therapies but the access to these treatments in the low-medium income countries remains a concern. Moreover, as observed in the data published at our center, the patients aged less than 30 years perform worse than those aged between 30 to 40 years (17). This could indicate aggressive disease biology that could plausibly be an area of research into personalized treatment of patients with YLC. Additionally, genome-wide gene-environment interaction studies could provide information on whether these patients have higher susceptibility to develop lung cancers due to differences in metabolism of environmental carcinogens.

The issues pertaining to oncofertility and sexual health are vital to be discussed with survivors as well as those on ongoing therapy. Effective management of treatment-related side effects are paramount because of their potential impact on QoL and financial well-being of patients with YLC. This subgroup of patients has added requirements of psychosocial support to enable them to cope with the effects of cytotoxic or targeted treatments.

There is a need for more multicenter collaborations to produce data on this rare and unique subset of patients. India and other Asian nations, with a larger proportion of younger patients can serve as important contributors to literature on YLC. Further Indian studies focusing on the YLC cohort will be vital in the near future for a better understanding of the germline mutational landscape, oncogenic driver analysis, treatment patterns and survival outcomes. The development of registries can pave the way for better molecular characterization, understanding of the exposome and disease biology, with the hope of uncovering newer therapeutic options in the YLC population.

## References

[1] Bray F Laversanne M Sung H Ferlay J Siegel RL Soerjomataram I. Global cancer statistics 2022: GLOBOCAN estimates of incidence and mortality worldwide for 36 cancers in 185 countries. CA: A Cancer J Clin. (2024) 74:229–63. doi: 10.3322/caac.21834, PMID: 38572751

[2] Hendriks LEL Remon J Faivre-Finn C Garassino MC Heymach JV Kerr KM. Non-small-cell lung cancer. Nat Rev Dis Primers. (2024) 10:71. doi: 10.1038/s41572-024-00551-9, PMID: 39327441

[3] Bravo-Iñiguez C Perez Martinez M Armstrong KW Jaklitsch MT. Surgical resection of lung cancer in the elderly. Thorac Surg Clin. (2014) 24:371–81. doi: 10.1016/j.thorsurg.2014.07.001, PMID: 25441130

[4] Li J Yang F Li X Zhang M Fu R Yin X. Characteristics, survival, and risk factors of Chinese young lung cancer patients: the experience from two institutions. Oncotarget. (2017) 8:89236–44. doi: 10.18632/oncotarget.19183, PMID: 29179515 PMC5687685

[5] Liu M Cai X Yu W Lv C Fu X. Clinical significance of age at diagnosis among young non-small cell lung cancer patients under 40 years old: a population-based study. Oncotarget. (2015) 6:44963–70. doi: 10.18632/oncotarget.5524, PMID: 26517681 PMC4792604

[6] Rich AL Khakwani A Free CM Tata LJ Stanley RA Peake MD. Non-small cell lung cancer in young adults: presentation and survival in the English National Lung Cancer Audit. QJM. (2015) 108:891–7. doi: 10.1093/qjmed/hcv052, PMID: 25725079

[7] Jemal A Ma J Rosenberg PS Siegel R Anderson WF. Increasing lung cancer death rates among young women in southern and midwestern states. J Clin Oncol. (2012) 30:2739–44. doi: 10.1200/JCO.2012.42.6098, PMID: 22734032 PMC3402885

[8] Zhang J Chen SF Zhen Y Xiang J Wu C Bao P. Multicenter analysis of lung cancer patients younger than 45 years in Shanghai. Cancer. (2010) 116:3656–62. doi: 10.1002/cncr.25100, PMID: 20564076

[9] Pal M Das D Pandey M. Understanding genetic variations associated with familial breast cancer. World J Surg Oncol. (2024) 22:271. doi: 10.1186/s12957-024-03553-9, PMID: 39390525 PMC11465949

[10] Rebuzzi F Ulivi P Tedaldi G. Genetic predisposition to colorectal cancer: how many and which genes to test? Int J Mol Sci. (2023) 24:2137. doi: 10.3390/ijms24032137, PMID: 36768460 PMC9916931

[11] Sacher AG Dahlberg SE Heng J Mach S Jänne PA Oxnard GR. Association between younger age and targetable genomic alterations and prognosis in non-small-cell lung cancer. JAMA Oncol. (2016) 2:313–20. doi: 10.1001/jamaoncol.2015.4482, PMID: 26720421 PMC4819418

[12] Singh N Agrawal S Jiwnani S Khosla D Malik PS Mohan A. Lung cancer in India. J Thorac Oncol. (2021) 16:1250–66. doi: 10.1016/j.jtho.2021.02.004, PMID: 34304854

[13] Statista. India - age distribution (2023). Available online at: https://www.statista.com/statistics/271315/age-distribution-in-India/ (Accessed January 12, 2025).

[14] Warren GW Cummings KM. Tobacco and lung cancer: risks, trends, and outcomes in patients with cancer. Am Soc Clin Oncol Educ Book. (2013) 33):359–64. doi: 10.14694/EdBook_AM.2013.33.359 23714547

[15] Hou H Zhu H Zhao H Yan W Wang Y Jiang M. Comprehensive molecular characterization of young Chinese patients with lung adenocarcinoma identified a distinctive genetic profile. Oncologist. (2018) 23:1008–15. doi: 10.1634/theoncologist.2017-0629, PMID: 29700208 PMC6192606

[16] Candal-Pedreira C Ruano-Ravina A Calvo de Juan V Cobo M Trigo JM Rodríguez-Abreu D. Comparison of clinical and genetic characteristics between younger and older lung cancer patients. Arch Bronconeumol. (2024) 60:88–94. doi: 10.1016/j.arbres.2023.12.005, PMID: 38160163

[17] Malik PS Pathak N Sharma A Birla M Rastogi A Sharma A. Young onset lung cancer in India: insights into clinical, demographic, and genomic profiles. Clin Lung Cancer. (2025) 26:420–428.e4. doi: 10.1016/j.cllc.2025.02.014, PMID: 40122770

[18] Asomaning K Miller DP Liu G Wain JC Lynch TJ Su L. Second hand smoke, age of exposure and lung cancer risk. Lung Cancer. (2008) 61:13–20. doi: 10.1016/j.lungcan.2007.11.013, PMID: 18191495 PMC2515267

[19] Grosche B Kreuzer M Kreisheimer M Schnelzer M Tschense A. Lung cancer risk among German male uranium miners: a cohort study, 1946-1998. Br J Cancer. (2006) 95:1280–7. doi: 10.1038/sj.bjc.6603403, PMID: 17043686 PMC2360564

[20] Gunani M Winayak R Agarwal A Ghose A Das R Prabhash K. Spotlight on lung cancer disparities in India. JCO Glob Oncol. (2025) 11:e2400327. doi: 10.1200/GO-24-00327, PMID: 39919262 PMC11892605

[21] Noronha V Pinninti R Patil VM Joshi A Prabhash K. Lung cancer in the Indian subcontinent. South Asian J Cancer. (2016) 5:95–103. doi: 10.4103/2278-330X.187571, PMID: 27606290 PMC4991146

[22] Liang H Zhou X Zhu Y Li D Jing D Su X. Association of outdoor air pollution, lifestyle, genetic factors with the risk of lung cancer: A prospective cohort study. Environ Res. (2023) 218:114996. doi: 10.1016/j.envres.2022.114996, PMID: 36481370

[23] Global Burden of Disease Study. Global, regional, and national comparative risk assessment of 84 behavioural, environmental and occupational, and metabolic risks or clusters of risks for 195 countries and territories, 1990–2017: a systematic analysis for the Global Burden of Disease Study 2017. Lancet. (2018) 392:1923–94. doi: 10.1016/S0140-6736(18)32225-6, PMID: 30496105 PMC6227755

[24] Hill W Lim EL Weeden CE Lee C Augustine M Chen K. Lung adenocarcinoma promotion by air pollutants. Nature. (2023) 616:159–67. doi: 10.1038/s41586-023-05874-3, PMID: 37020004 PMC7614604

[25] IARC Working Group. Outdoor air pollution. IARC Monogr Eval Carcinog Risks Hum. (2016) 109:9–444. 29905447 PMC7682275

[26] Katpattil SS. Domestic cooking fuel as a risk factor for lung cancer in women: A case control study. Ann Oncol. (2017) 28:ii6. doi: 10.1093/annonc/mdx087.003

[27] Jaiswal VB Meshram PU. Behavioral Change in Determinants of the Choice of Fuels amongst Rural Households after the Introduction of Clean Fuel Program: A District-Level Case Study. Glob Chall. (2020) 5:2000004. doi: 10.1002/gch2.202000004, PMID: 33552549 PMC7857130

[28] Iyer H Ghosh T Garg A Agarwal H Jain D Pandey R. Lung cancer in Asian Indian females: Identification of disease-specific characteristics and outcome measures over a 12-year period. Lung India. (2023) 40:4–11. doi: 10.4103/lungindia.lungindia_43_22, PMID: 36695252 PMC9894289

[29] Long E Patel H Byun J Amos CI Choi J. Functional studies of lung cancer GWAS beyond association. Hum Mol Genet. (2022) 31:R22–36. doi: 10.1093/hmg/ddac140, PMID: 35776125 PMC9585683

[30] Zhang R Chu M Zhao Y Wu C Guo H Shi Y. A genome-wide gene-environment interaction analysis for tobacco smoke and lung cancer susceptibility. Carcinogenesis. (2014) 35:1528–35. doi: 10.1093/carcin/bgu076, PMID: 24658283 PMC4076813

[31] Wei S Wang LE McHugh MK Han Y Xiong M Amos CI. Genome-wide gene-environment interaction analysis for asbestos exposure in lung cancer susceptibility. Carcinogenesis. (2012) 33:1531–7. doi: 10.1093/carcin/bgs188, PMID: 22637743 PMC3499061

[32] Liu CY Stücker I Chen C Goodman G McHugh MK D’Amelio AM. Genome-wide gene-asbestos exposure interaction association study identifies a common susceptibility variant on 22q13.31 associated with lung cancer risk. Cancer Epidemiol Biomarkers Prev. (2015) 24:1564–73. doi: 10.1158/1055-9965.EPI-15-0021, PMID: 26199339 PMC4592421

[33] McKay JD Hung RJ Gaborieau V Boffetta P Chabrier A Byrnes G. Lung cancer susceptibility locus at 5p15.33. Nat Genet. (2008) 40:1404–6. doi: 10.1038/ng.254, PMID: 18978790 PMC2748187

[34] Zhang R Shen S Wei Y Zhu Y Li Y Chen J. A large-scale genome-wide gene-gene interaction study of lung cancer susceptibility in Europeans with a trans-ethnic validation in Asians. J Thorac Oncol. (2022) 17:974–90. doi: 10.1016/j.jtho.2022.04.011, PMID: 35500836 PMC9512697

[35] McKay JD Hung RJ Han Y Zong X Carreras-Torres R Christiani DC. Large-scale association analysis identifies new lung cancer susceptibility loci and heterogeneity in genetic susceptibility across histological subtypes. Nat Genet. (2017) 49:1126–32. doi: 10.1038/ng.3892, PMID: 28604730 PMC5510465

[36] Rachtan J Sokołowski A Niepsuj S Zemła B Zwierko M. Familial lung cancer risk among women in Poland. Lung Cancer. (2009) 65:138–43. doi: 10.1016/j.lungcan.2008.10.029, PMID: 19084289

[37] Parry EM Gable DL Stanley SE Khalil SE Antonescu V Florea L. Germline mutations in DNA repair genes in lung adenocarcinoma. J Thorac Oncol. (2017) 12:1673–8. doi: 10.1016/j.jtho.2017.08.011, PMID: 28843361 PMC5659909

[38] Coté ML Liu M Bonassi S Neri M Schwartz AG Christiani DC. Increased risk of lung cancer in individuals with a family history of the disease: A pooled analysis from the International Lung Cancer Consortium. Eur J Cancer. (2012) 48:1957–68. doi: 10.1016/j.ejca.2012.01.038, PMID: 22436981 PMC3445438

[39] Sorscher S LoPiccolo J Heald B Chen E Bristow SL Michalski ST. Rate of pathogenic germline variants in patients with lung cancer. JCO Precis Oncol. (2023) 7:e2300190. doi: 10.1200/PO.23.00190, PMID: 37992258 PMC10681406

[40] Wei B Zhao J Li J Feng J Sun M Wang Z. Pathogenic germline variants in BRCA1 and TP53 increase lung cancer risk in Chinese. Cancer Med. (2023) 12:21219–28. doi: 10.1002/cam4.6692, PMID: 37930190 PMC10726856

[41] Donner I Katainen R Sipilä LJ Aavikko M Pukkala E Aaltonen LA. Germline mutations in young non-smoking women with lung adenocarcinoma. Lung Cancer. (2018) 122:76–82. doi: 10.1016/j.lungcan.2018.05.027, PMID: 30032850

[42] Caron O Frebourg T Benusiglio PR Foulon S Brugières L. Lung adenocarcinoma as part of the li-fraumeni syndrome spectrum: preliminary data of the LIFSCREEN randomized clinical trial. JAMA Oncol. (2017) 3:1736–7. doi: 10.1001/jamaoncol.2017.1358, PMID: 28772306 PMC5824279

[43] Reckamp KL Behrendt CE Slavin TP Gray SW Castillo DK Koczywas M. Germline mutations and age at onset of lung adenocarcinoma. Cancer. (2021) 127:2801–6. doi: 10.1002/cncr.33573, PMID: 33858029 PMC8794435

[44] Rastogi A Malik PS Chitikela SD Rathor A Gupta I Pramanik R. 190P Prevalence of germline variants in Indian non-small cell lung cancer patients with family history of cancer. ESMO Open. (2024) 9. doi: 10.1016/j.esmoop.2024.102764

[45] Schwartz AG Yang P Swanson GM. Familial risk of lung cancer among nonsmokers and their relatives. Am J Epidemiol. (1996) 144:554–62. doi: 10.1093/oxfordjournals.aje.a008965, PMID: 8797515

[46] Kreuzer M Kreienbrock L Gerken M Heinrich J Bruske-Hohlfeld I Muller KM. Risk factors for lung cancer in young adults. Am J Epidemiol. (1998) 147:1028–37. doi: 10.1093/oxfordjournals.aje.a009396, PMID: 9620046

[47] Schwartz AG Siegfried JM Weiss L. Familial aggregation of breast cancer with early onset lung cancer. Genet Epidemiol. (1999) 17:274–84. doi: 10.1002/(SICI)1098-2272(199911)17:4<274::AID-GEPI3>3.0.CO;2-A 10520210

[48] Hemminki K Li X. Familial risk for lung cancer by histology and age of onset: evidence for recessive inheritance. Exp Lung Res. (2005) 31:205–15. doi: 10.1080/01902140490495606, PMID: 15824021

[49] Coté ML Kardia SLR Wenzlaff AS Ruckdeschel JC Schwartz AG. Risk of lung cancer among white and black relatives of individuals with early-onset lung cancer. JAMA. (2005) 293:3036–42. doi: 10.1001/jama.293.24.3036, PMID: 15972566

[50] Cassidy A Myles JP van Tongeren M Page RD Liloglou T Duffy SW. The LLP risk model: an individual risk prediction model for lung cancer. Br J Cancer. (2008) 98:270–6. doi: 10.1038/sj.bjc.6604158, PMID: 18087271 PMC2361453

[51] Taioli E Pedotti P. Pooled analysis on metabolic gene polymorphisms and lung cancer. Exp Lung Res. (2005) 31:217–22. doi: 10.1080/01902140490495615, PMID: 15824022

[52] Coté ML Kardia SLR Wenzlaff AS Land SJ Schwartz AG. Combinations of glutathione S -transferase genotypes and risk of early-onset lung cancer in Caucasians and African Americans: a population-based study. Carcinogenesis. (2005) 26:811–9. doi: 10.1093/carcin/bgi023, PMID: 15661806

[53] Landi S Gemignani F Canzian F Gaborieau V Barale R Landi D. DNA repair and cell cycle control genes and the risk of young-onset lung cancer. Cancer Res. (2006) 66:11062–9. doi: 10.1158/0008-5472.CAN-06-1039, PMID: 17108146

[54] Gemignani F Landi S Szeszenia-Dabrowska N Zaridze D Lissowska J Rudnai P. Development of lung cancer before the age of 50: the role of xenobiotic metabolizing genes. Carcinogenesis. (2007) 28:1287–93. doi: 10.1093/carcin/bgm021, PMID: 17259654

[55] Rosenberger A Illig T Korb K Klopp N Zietemann V Wölke G. Do genetic factors protect for early onset lung cancer? A case control study before the age of 50 years. BMC Cancer. (2008) 8:60. doi: 10.1186/1471-2407-8-60, PMID: 18298806 PMC2292731

[56] An Y Jin G Wang H Wang Y Liu H Li R. Polymorphisms in hMLH1 and risk of early-onset lung cancer in a southeast Chinese population. Lung Cancer. (2008) 59:164–70. doi: 10.1016/j.lungcan.2007.08.003, PMID: 17870204

[57] Sauter W Rosenberger A Beckmann L Kropp S Mittelstrass K Timofeeva M. Matrix metalloproteinase 1 (MMP1) is associated with early-onset lung cancer. Cancer Epidemiol Biomarkers Prev. (2008) 17:1127–35. doi: 10.1158/1055-9965.EPI-07-2840, PMID: 18483334

[58] Graziano C Comin CE Crisci C Novelli L Politi L Messerini L. Functional polymorphisms of the microsomal epoxide hydrolase gene: A reappraisal on a early-onset lung cancer patients series. Lung Cancer. (2009) 63:187–93. doi: 10.1016/j.lungcan.2008.05.004, PMID: 18571762

[59] Thomas A Chen Y Yu T Jakopovic M Giaccone G. Trends and characteristics of young non-small cell lung cancer patients in the United States. Front Oncol. (2015) 5. doi: 10.3389/fonc.2015.00113, PMID: 26075181 PMC4443720

[60] Suidan AM Roisman L Belilovski Rozenblum A Ilouze M Dudnik E Zer A. Lung cancer in young patients: higher rate of driver mutations and brain involvement, but better survival. J Glob Oncol. (2019) 5:1–8. doi: 10.1200/JGO.18.00216, PMID: 31067141 PMC6550091

[61] Subramanian J Morgensztern D Goodgame B Baggstrom MQ Gao F Piccirillo J. Distinctive characteristics of non-small cell lung cancer (NSCLC) in the young: a surveillance, epidemiology, and end results (SEER) analysis. J Thorac Oncol. (2010) 5:23–8. doi: 10.1097/JTO.0b013e3181c41e8d, PMID: 19934774

[62] Vashistha V Garg A Iyer H Jain D Madan K Hadda V. A comprehensive comparison between young and older-age non-small cell lung cancer patients at a public referral centre in Delhi, India. Ecancermedicalscience. (2021) 15:1223. doi: 10.3332/ecancer.2021.1223, PMID: 34158827 PMC8183650

[63] Sekine I Nishiwaki Y Yokose T Nagai K Suzuki K Kodama T. Young lung cancer patients in Japan: different characteristics between the sexes. Ann Thorac Surg. (1999) 67:1451–5. doi: 10.1016/S0003-4975(99)00171-X, PMID: 10355430

[64] Liu B Quan X Xu C Lv J Li C Dong L. Lung cancer in young adults aged 35 years or younger: A full-scale analysis and review. J Cancer. (2019) 10:3553–9. doi: 10.7150/jca.27490, PMID: 31293660 PMC6603399

[65] Hughes DJ Kapiris M Podvez Nevajda A McGrath H Stavraka C Ahmad S. Non-small cell lung cancer (NSCLC) in young adults, age < 50, is associated with late stage at presentation and a very poor prognosis in patients that do not have a targeted therapy option: A real-world study. Cancers. (2022) 14:6056. doi: 10.3390/cancers14246056, PMID: 36551542 PMC9776398

[66] Dong Y Zhou S Li J Zhang Y Che G. Distant metastatic patterns in young and old non-small cell lung cancer patients: A dose–response analysis based on SEER population. Heliyon. (2024) 10:e36657. doi: 10.1016/j.heliyon.2024.e36657, PMID: 39281448 PMC11401064

[67] Mohan A Garg A Gupta A Sahu S Choudhari C Vashistha V. Clinical profile of lung cancer in North India: A 10-year analysis of 1862 patients from a tertiary care center. Lung India. (2020) 37:190–7. doi: 10.4103/lungindia.lungindia_333_19, PMID: 32367839 PMC7353932

[68] Chauhan A Parmar M Dash GC Solanki H Chauhan S Sharma J. The prevalence of tuberculosis infection in India: A systematic review and meta-analysis. Indian J Med Res. (2023) 157:135–51. doi: 10.4103/ijmr.ijmr_382_23, PMID: 37202933 PMC10319385

[69] Ramachandran K Thankagunam B Karuppusami R Christopher DJ. Physician related delays in the diagnosis of lung cancer in India. J Clin Diagn Res. (2016) 10:OC05–8. doi: 10.7860/JCDR/2016/22737.8823, PMID: 28050418 PMC5198371

[70] Chandra S Mohan A Guleria R Singh V Yadav P. Delays during the diagnostic evaluation and treatment of lung cancer. Asian Pac J Cancer Prev. (2009) 10:453–6. 19640190

[71] DeCaro L Benfield JR. Lung cancer in young persons. J Thorac Cardiovasc Surg. (1982) 83:372–6. doi: 10.1016/S0022-5223(19)37271-X, PMID: 6278230

[72] Nugent WC Edney MT Hammerness PG Dain BJ Maurer LH Rigas JR. Non-small cell lung cancer at the extremes of age: impact on diagnosis and treatment. Ann Thorac Surg. (1997) 63:193–7. doi: 10.1016/s0003-4975(96)00745-x, PMID: 8993264

[73] Etzel CJ Lu M Merriman K Liu M Vaporciyan A Spitz MR. An epidemiologic study of early onset lung cancer. Lung Cancer. (2006) 52:129–34. doi: 10.1016/j.lungcan.2005.11.018, PMID: 16564601

[74] Hsu CL Chen KY Shih JY Ho CC Yang CH Yu CJ. Advanced non-small cell lung cancer in patients aged 45 years or younger: outcomes and prognostic factors. BMC Cancer. (2012) 12:241. doi: 10.1186/1471-2407-12-241, PMID: 22695392 PMC3420246

[75] Arnold BN Thomas DC Rosen JE Salazar MC Blasberg JD Boffa DJ. Lung cancer in the very young: treatment and survival in the national cancer data base. J Thorac Oncol. (2016) 11:1121–31. doi: 10.1016/j.jtho.2016.03.023, PMID: 27103511

[76] Hsu CH Tseng CH Chiang CJ Hsu KH Tseng JS Chen KC. Characteristics of young lung cancer: Analysis of Taiwan’s nationwide lung cancer registry focusing on epidermal growth factor receptor mutation and smoking status. Oncotarget. (2016) 7:46628–35. doi: 10.18632/oncotarget.9338, PMID: 27191886 PMC5216823

[77] Vavalà T Monica V Lo Iacono M Mele T Busso S Righi L. Precision medicine in age-specific non-small-cell-lung-cancer patients: Integrating biomolecular results into clinical practice-A new approach to improve personalized translational research. Lung Cancer. (2017) 107:84–90. doi: 10.1016/j.lungcan.2016.05.021, PMID: 27346245

[78] Tanaka K Hida T Oya Y Yoshida T Shimizu J Mizuno T. Unique prevalence of oncogenic genetic alterations in young patients with lung adenocarcinoma. Cancer. (2017) 123:1731–40. doi: 10.1002/cncr.30539, PMID: 28177518

[79] Luo W Tian P Wang Y Xu H Chen L Tang C. Characteristics of genomic alterations of lung adenocarcinoma in young never-smokers. Int J Cancer. (2018) 143:1696–705. doi: 10.1002/ijc.31542, PMID: 29667179 PMC6175072

[80] Giordano M Boldrini L Servadio A Niccoli C Melfi F Lucchi M. Differential microRNA expression profiles between young and old lung adenocarcinoma patients. Am J Transl Res. (2018) 10:892–900., PMID: 29636879 PMC5883130

[81] Yang B Li J Li F Zhou H Shi W Shi H. Comprehensive analysis of age-related somatic mutation profiles in Chinese young lung adenocarcinoma patients. Cancer Med. (2019) 8:1350–8. doi: 10.1002/cam4.1839, PMID: 30821106 PMC6488136

[82] Chen L Hu X Wu H Liu J Mu X Wu H. Unique profiles of targetable genomic alterations and prognosis in young Chinese patients with lung adenocarcinoma. Pathol Res Pract. (2019) 215:152407. doi: 10.1016/j.prp.2019.03.035, PMID: 30962004

[83] Yang S Song Z Cheng G. Genomic alterations and survival in young patients aged under 40 years with completely resected non-small cell lung cancer. Ann Transl Med. (2019) 7:140–0. doi: 10.21037/atm.2019.03.39, PMID: 31157261 PMC6511547

[84] Hou H Zhang C Qi X Zhou L Liu D Lv H. Distinctive targetable genotypes of younger patients with lung adenocarcinoma: a cBioPortal for cancer genomics data base analysis. Cancer Biol Ther. (2020) 21:26–33. doi: 10.1080/15384047.2019.1665392, PMID: 31594446 PMC7012066

[85] Catania C Botteri E Barberis M Conforti F Toffalorio F De Marinis F. Molecular features and clinical outcome of lung Malignancies in very young people. Future Oncol. (2015) 11:1211–21. doi: 10.2217/fon.15.10, PMID: 25832878

[86] Awad MM Oxnard GR Jackman DM Savukoski DO Hall D Shivdasani P. MET exon 14 mutations in non–small-cell lung cancer are associated with advanced age and stage-dependent MET genomic amplification and c-met overexpression. JCO. (2016) 34:721–30. doi: 10.1200/JCO.2015.63.4600, PMID: 26729443

[87] He CH Shih JF Lai SL Chen YM. Non–small cell lung cancer in the very young: Higher EGFR/ALK mutation proportion than the elder. J Chin Med Assoc. (2020) 83:461. doi: 10.1097/JCMA.0000000000000311, PMID: 32221155 PMC13048176

[88] Sugiyama E Goto K Ishii G Umemura S Yoh K Niho S. Higher incidence of EGFR exon 19 deletion in younger (age 40 or younger) patients with adenocarcinoma of the lung. JCO. (2012) 30:7044–4. doi: 10.1200/jco.2012.30.15_suppl.7044

[89] Tian Y Ma R Zhao W Wang S Zhou C Wu W. Comprehensive characterization of early-onset lung cancer, in Chinese young adults. Nat Commun. (2025) 16:1976. doi: 10.1038/s41467-025-57309-4, PMID: 40000630 PMC11861273

[90] Wu SG Liu YN Yu CJ Yang JCH Shih JY. Driver mutations of young lung adenocarcinoma patients with Malignant pleural effusion. Genes Chromosomes Cancer. (2018) 57:513–21. doi: 10.1002/gcc.22647, PMID: 30107055

[91] Ye T Pan Y Wang R Hu H Zhang Y Li H. Analysis of the molecular and clinicopathologic features of surgically resected lung adenocarcinoma in patients under 40 years old. J Thorac Dis. (2014) 6:1396–402. doi: 10.3978/j.issn.2072-1439.2014.08.50, PMID: 25364516 PMC4215147

[92] Wu X Zhao J Yang L Nie X Wang Z Zhang P. Next-generation sequencing reveals age-dependent genetic underpinnings in lung adenocarcinoma. J Cancer. (2022) 13:1565–72. doi: 10.7150/jca.65370, PMID: 35371328 PMC8965110

[93] Paik PK Felip E Veillon R Sakai H Cortot AB Garassino MC. Tepotinib in non–small-cell lung cancer with MET exon 14 skipping mutations. New Engl J Med. (2020) 383:931–43. doi: 10.1056/NEJMoa2004407, PMID: 32469185 PMC8422679

[94] Wolf J Seto T Han JY Reguart N Garon EB Groen HJM. Capmatinib in MET exon 14–mutated or MET-amplified non–small-cell lung cancer. New Engl J Med. (2020) 383:944–57. doi: 10.1056/NEJMoa2002787, PMID: 32877583

[95] Sharma S Noronha V Yadav A Mandhania M Mohanty SK Katara R. Comprehensive genomic profiling of Indian patients with lung cancer. JCO Glob Oncol. (2025) 11:e2400587. doi: 10.1200/GO-24-00587, PMID: 40324118

[96] Soria JC Ohe Y Vansteenkiste J Reungwetwattana T Chewaskulyong B Lee KH. Osimertinib in untreated EGFR-mutated advanced non-small-cell lung cancer. N Engl J Med. (2018) 378:113–25. doi: 10.1056/NEJMoa1713137, PMID: 29151359

[97] Mok TS Wu YL Ahn MJ Garassino MC Kim HR Ramalingam SS. Osimertinib or platinum-pemetrexed in EGFR T790M-positive lung cancer. N Engl J Med. (2017) 376:629–40. doi: 10.1056/NEJMoa1612674, PMID: 27959700 PMC6762027

[98] Shaw AT Bauer TM de Marinis F Felip E Goto Y Liu G. First-line lorlatinib or crizotinib in advanced ALK-positive lung cancer. New Engl J Med. (2020) 383:2018–29. doi: 10.1056/NEJMoa2027187, PMID: 33207094

[99] Mok TSK Wu YL Kudaba I Kowalski DM Cho BC Turna HZ. Pembrolizumab versus chemotherapy for previously untreated, PD-L1-expressing, locally advanced or metastatic non-small-cell lung cancer (KEYNOTE-042): a randomised, open-label, controlled, phase 3 trial. Lancet. (2019) 393:1819–30. doi: 10.1016/S0140-6736(18)32409-7, PMID: 30955977

[100] Reck M Rodríguez–Abreu D Robinson AG Hui R Csőszi T Fülöp A. Updated analysis of KEYNOTE-024: pembrolizumab versus platinum-based chemotherapy for advanced non–small-cell lung cancer with PD-L1 tumor proportion score of 50% or greater. JCO. (2019) 37:537–46. doi: 10.1200/JCO.18.00149, PMID: 30620668

[101] Voruganti T Soulos PR Mamtani R Presley CJ Gross CP. Association between age and survival trends in advanced non–small cell lung cancer after adoption of immunotherapy. JAMA Oncol. (2023) 9:334–41. doi: 10.1001/jamaoncol.2022.6901, PMID: 36701150 PMC9880865

[102] Shyam Sunder S Sharma UC Pokharel S. Adverse effects of tyrosine kinase inhibitors in cancer therapy: pathophysiology, mechanisms and clinical management. Signal Transduct Target Ther. (2023) 8:262. doi: 10.1038/s41392-023-01469-6, PMID: 37414756 PMC10326056

[103] Tseng LC Chen KH Wang CL Weng LC. Effects of tyrosine kinase inhibitor therapy on skin toxicity and skin-related quality of life in patients with lung cancer: An observational study. Med (Baltimore). (2020) 99:e20510. doi: doi: 10.1097/MD.0000000000020510, PMID: 32501998 PMC7306373

[104] Lustberg MB Kuderer NM Desai A Bergerot C Lyman GH. Mitigating long-term and delayed adverse events associated with cancer treatment: implications for survivorship. Nat Rev Clin Oncol. (2023) 20:527–42. doi: 10.1038/s41571-023-00776-9, PMID: 37231127 PMC10211308

[105] Florez N Kiel L Horiguchi M Kaufman R Haradon D Sanchez M. Young lung cancer: Psychosocial needs assessment. JCO. (2024) 42:12104–4. doi: 10.1200/JCO.2024.42.16_suppl.12104

[106] Lambertini M Peccatori FA Demeestere I Amant F Wyns C Stukenborg JB. Fertility preservation and post-treatment pregnancies in post-pubertal cancer patients: ESMO Clinical Practice Guidelines†. Ann Oncol. (2020) 31:1664–78. doi: 10.1016/j.annonc.2020.09.006, PMID: 32976936

[107] Rambhatla A Strug MR De Paredes JG Cordoba Munoz MI Thakur M. Fertility considerations in targeted biologic therapy with tyrosine kinase inhibitors: a review. J Assist Reprod Genet. (2021) 38:1897–908. doi: 10.1007/s10815-021-02181-6, PMID: 33826052 PMC8417172

[108] Vandecaveye V Amant F Lecouvet F Van Calsteren K Dresen RC. Imaging modalities in pregnant cancer patients. Int J Gynecol Cancer. (2021) 31:423–31. doi: 10.1136/ijgc-2020-001779, PMID: 33649009 PMC7925814

[109] Basta P Bak A Roszkowski K. Cancer treatment in pregnant women. Contemp Oncol (Pozn). (2015) 19:354–60. doi: 10.5114/wo.2014.46236, PMID: 26793018 PMC4709394

[110] Koutras A Ntounis T Fasoulakis Z Papalios T Pittokopitou S Prokopakis I. Cancer treatment and immunotherapy during pregnancy. Pharmaceutics. (2022) 14:2080. doi: 10.3390/pharmaceutics14102080, PMID: 36297515 PMC9611953

[111] Peccatori FA Azim HA Orecchia R Hoekstra HJ Pavlidis N Kesic V. Cancer, pregnancy and fertility: ESMO Clinical Practice Guidelines for diagnosis, treatment and follow-up. Ann Oncol. (2013) 24 Suppl 6:vi160–170. doi: 10.1093/annonc/mdt199, PMID: 23813932

[112] Dagogo-Jack I Gainor JF Porter RL Schultz KR Solomon BJ Stevens S. Clinicopathologic features of NSCLC diagnosed during pregnancy or the peripartum period in the era of molecular genotyping. J Thorac Oncol. (2016) 11:1522–8. doi: 10.1016/j.jtho.2016.05.031, PMID: 27296107 PMC5002360

[113] Simons E Camidge DR. Lung cancer oncogene-directed therapy, fertility, and pregnancy. J Thorac Oncol. (2024) 19:866–76. doi: 10.1016/j.jtho.2024.01.003, PMID: 38185202

